# Whole-Genome Characterization of Pediatric Urinary *E. coli* Isolates from a Tertiary Care Pediatric Hospital in Guayaquil, Ecuador: Identification of the High-Risk ST1193 Clone

**DOI:** 10.3390/antibiotics15100996

**Published:** 2026-10-08

**Authors:** Laura Lapo-Calle, Isaías Mejía-Limones, Ricardo David Avellán-Llaguno, Julio Cesar Hidalgo-Olmedo, Juan Gregorio Chang-Asinc, Gabriel Morey-León, Derly Andrade-Molina

**Affiliations:** 1 Laboratorio de Ciencias Ómicas, Facultad de Ciencias de La Salud, Universidad Espíritu Santo, Samborondón 092301, Ecuador; laura.lapo@uees.edu.ec (L.L.-C.); imejia@uees.edu.ec (I.M.-L.); ricardo.avellan@uees.edu.ec (R.D.A.-L.); 2 Hospital de Niños Dr. Roberto Gilbert E. Junta de Beneficencia de Guayaquil, Guayaquil 090514, Ecuador; jhidalgol@jbgye.org.ec (J.C.H.-O.); jchanga@jbgye.org.ec (J.G.C.-A.); 3 Facultad de Ciencias de la Salud y Desarrollo Humano, Universidad Ecotec, Km. 13.5 Samborondón, Samborondón EC092302, Ecuador

**Keywords:** *E. coli*, urinary tract infections (UTIs), pediatric, high-risk clone, whole-genome sequencing, Ecuador

## Abstract

Background: *Escherichia coli* is a leading cause of urinary tract infections (UTIs) in children, and increasing antimicrobial resistance complicates empirical treatment. However, genomic information on pediatric urinary *E. coli* associated with urinary tract infection in Ecuador remains limited. Objective: This exploratory study analyzed four *E. coli* isolates obtained from urine samples of pediatric patients at a tertiary hospital in Guayaquil, Ecuador. Methods: Whole-genome sequencing (WGS) was performed to determine sequence types (STs), serotypes, antimicrobial resistance genes (ARGs) virulence factors (VFs), and phylogenetic relationships. Results: Two isolates were assigned to the globally disseminated high-risk clone ST1193 and carried extended-spectrum β-lactamase (ESBL) genes, including *bla_CTX-M-27_*, whereas one isolate was assigned to ST162 and carried *bla_CTX-M-65_*. The remaining isolate could not be assigned to a complete ST because of missing MLST loci, but it harbored *bla_CTX-M-15_*, *bla_OXA-1_*, and *bla_TEM-1_*. Fluoroquinolone resistance-associated substitutions were detected in *gyr*A, *par*C, and *par*E in three isolates. The isolates shared a conserved set of VFs involved in adhesion, iron acquisition, and secretion systems. ARGs were frequently associated with insertion sequences and IncF plasmid replicons, indicating a potential contribution of mobile genetic elements (MGEs) to the dissemination of antimicrobial resistance. Conclusions: Despite the limited number of isolates, these findings provide preliminary genomic evidence of the presence of ST1193 and diverse ESBL-producing urinary *E. coli* lineages among pediatric patients in Guayaquil, supporting the need for expanded genomic surveillance and antimicrobial stewardship efforts at the hospital level.

## 1. Introduction

*E*. *coli* is a Gram-negative bacterium that forms part of the normal intestinal microbiota in humans and warm-blooded animals, contributing to gut homeostasis [1]. However, some strains have acquired VFs that enable them to cause intestinal and extraintestinal infections [2]. In addition to virulence, pathogenic *E. coli* has increasingly acquired ARGs conferring resistance to multiple antimicrobial classes, including resistance to third-generation cephalosporins, primarily mediated by the production of extended-spectrum β-lactamases (ESBLs) and AmpC β-lactamases, which hydrolyze beta-lactam antibiotics and compromise therapeutic efficacy [3].

Uropathogenic *E. coli* (UPEC), a subtype of ExPEC, is responsible for the majority of UTIs, particularly in pediatric populations [4,5,6,7]. UPEC strains are genetically heterogeneous and may harbor diverse combinations of VFs, including those involved in adhesion, iron uptake, immune evasion, toxin production, and survival within the urinary tract [8,9,10]. Among globally disseminated extraintestinal *E. coli* lineages, sequence type ST1193 has emerged as a clinically relevant high-risk clone [11]. Notably, ST1193 has demonstrated a pattern of rapid international expansion and dissemination comparable to that previously observed for ST131, suggesting its emergence as another globally successful ExPEC lineage. ST1193 is associated with phylogroup B2, which frequently exhibits fluoroquinolone resistance and ESBL production, while carbapenemase-encoding genes have also been reported in some isolates [12,13]. Importantly, ST1193 has been implicated in maternal and neonatal infections, highlighting its clinical relevance in obstetric and pediatric settings [14].

In Ecuador, several studies have investigated *E. coli* isolates from clinical and non-clinical settings. Recent genomic surveillance has predominantly focused on the One Health interface. Most sequence-based studies have characterized isolates from domestic and food-producing animals, environmental sources such as urban rivers, and diarrheagenic strains causing gastrointestinal infections [15,16,17]. In contrast, local literature addressing pediatric urinary *E. coli* in pediatric patients has been largely limited to phenotypic antimicrobial resistance profiling and clinical descriptions. Retrospective pediatric studies from Quito and Cuenca identify *E. coli* as the predominant uropathogen (up to 90.6% of cases), with high resistance rates to first-line antibiotics such as ampicillin (up to 92%) and trimethoprim-sulfamethoxazole determined by standard antibiograms [18,19]. Similarly, recent surveillance in coastal regions relies on automated phenotypic testing, reporting high resistance to β-lactamase inhibitors [20]. Genomic information on pediatric urinary *E. coli* isolates in Ecuador remains limited, particularly regarding high-risk sequence types, antimicrobial resistance determinants, virulence-associated genes, mobile genetic elements, and their phylogenetic context.

In this study, we performed whole-genome sequencing (WGS) of four ESBL-producing *E. coli* isolates recovered from urine samples of pediatric patients at a tertiary hospital in Guayaquil, Ecuador. We characterized their sequence types (STs), serotypes, antimicrobial resistance genes, virulence-associated genes, plasmid replicons, mobile genetic elements, and phylogenetic relationships to provide exploratory genomic information on pediatric urinary *E. coli* isolates recovered at a tertiary pediatric hospital in Guayaquil, Ecuador.

## 2. Results

### 2.1. Antimicrobial Susceptibility Profiles

Phenotypic antimicrobial susceptibility data were available for EC8CRG, EC27CRG, and EC38CRG; data were unavailable for EC22CRG. All three isolates with available results showed resistance to all six antibiotics tested (AMP, SAM, CEF, CIP, GEN, and SXT), which belong to three different classes, indicating a multidrug-resistant phenotype (Supplementary Table S1).

### 2.2. Molecular Features of E. coli Isolates

The four genome assemblies had an average size of 4.95 Mbp and a mean N50 of 6.92 kbp, with an average of 1430 contigs per assembly (Table 1). Genome completeness exceeded 95% for all isolates, with estimated contamination ranging from 0,49% to 3.05%. Despite this overall completeness, the high degree of assembly fragmentation limited the recovery of complete Multilocus sequence typing (MLST) profiles in one isolate and constrained the interpretation of genomic contexts involving ARGs and MGEs. ANI values above 98.5% relative to *E. coli* reference genomes supported species-level identification of all four isolates.

MLST assigned EC22CRG and EC8CRG to ST1193 and the EC27CRG isolate to ST162. For EC38CRG isolate, a complete sequence type could not be assigned because the *icd* locus and the *mdh* allele required for the Achtman scheme were not recovered. Nevertheless, partial allele matches were identified, with the closest profiles corresponding to ST69 (Supplementary Table S2).

In EC27CRG, the *icd* locus showed a single-nucleotide difference from the reference *icd_1* allele. Phylogroup assignment using the Clermont scheme identified isolates as belonging to phylogroups A, B1, and B2. Serotype determination by ECTyper revealed three O:H combinations among the strains, with O75:H5 being present in 2 of the 4 isolates. (Table 1).

#### 2.2.1. Resistome

**Figure 1:**
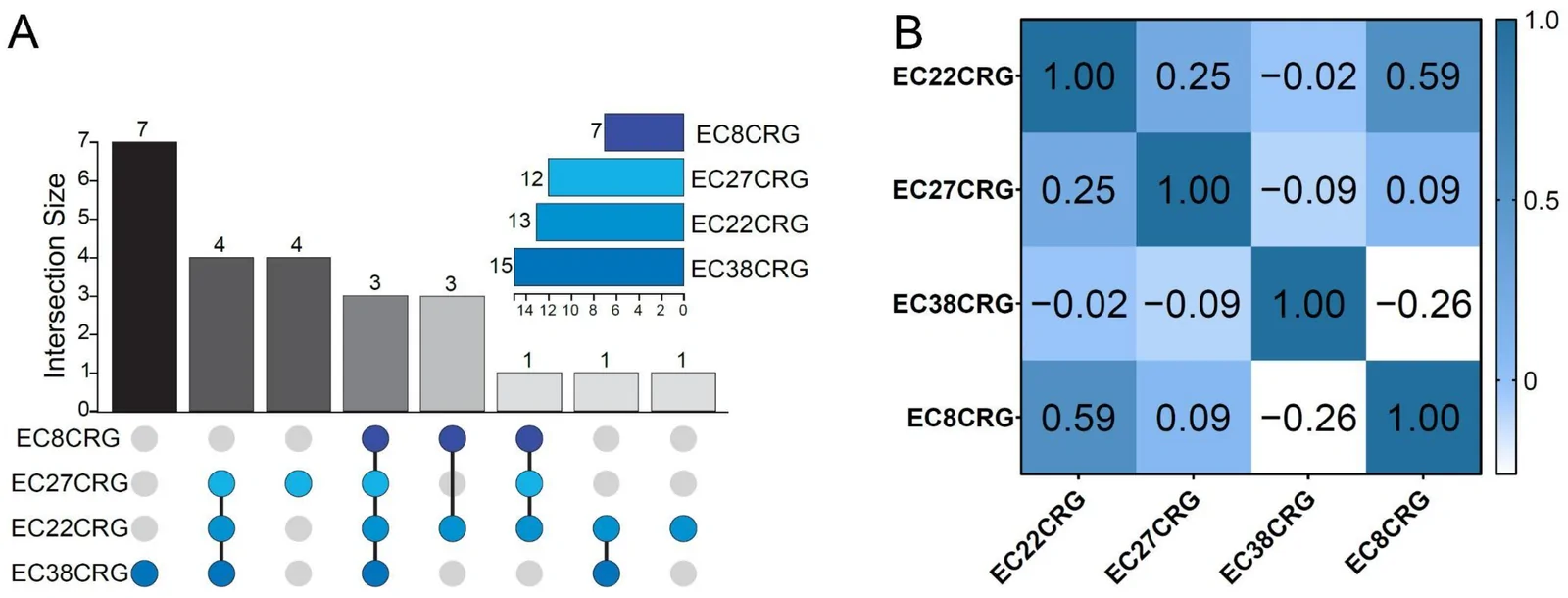
Comparative resistome profiling among *E. coli* clinical isolates. (**A**) Upset plot of conserved and strain-specific ARGs. (**B**) Pairwise similarity matrix showing the proportion of shared resistance determinants.

Comparative resistome analysis identified 24 ARGs distributed across four isolates. EC38CRG harbored the most (*n* = 15). Comparative analysis identified a conserved core resistome shared across all isolates, including *aadA5*, *dfrA17*, and *sul1*, alongside strain-specific ARGs that contributed to the variability of the accessory resistome, as illustrated by the UpSet plot and similarity matrix (Figure 1A). Notably, EC38CRG showed the broadest functional multidrug resistance profile, encompassing determinants conferring resistance to β-lactams, aminoglycosides, fluoroquinolones, trimethoprim, sulfonamides, tetracyclines, and phenicols (Figure 1A). Consistent with this broader ARG repertoire, EC38CRG showed the lowest resistome similarity to the remaining isolates in the pairwise similarity analysis (Figure 1B).

**Figure 2:**
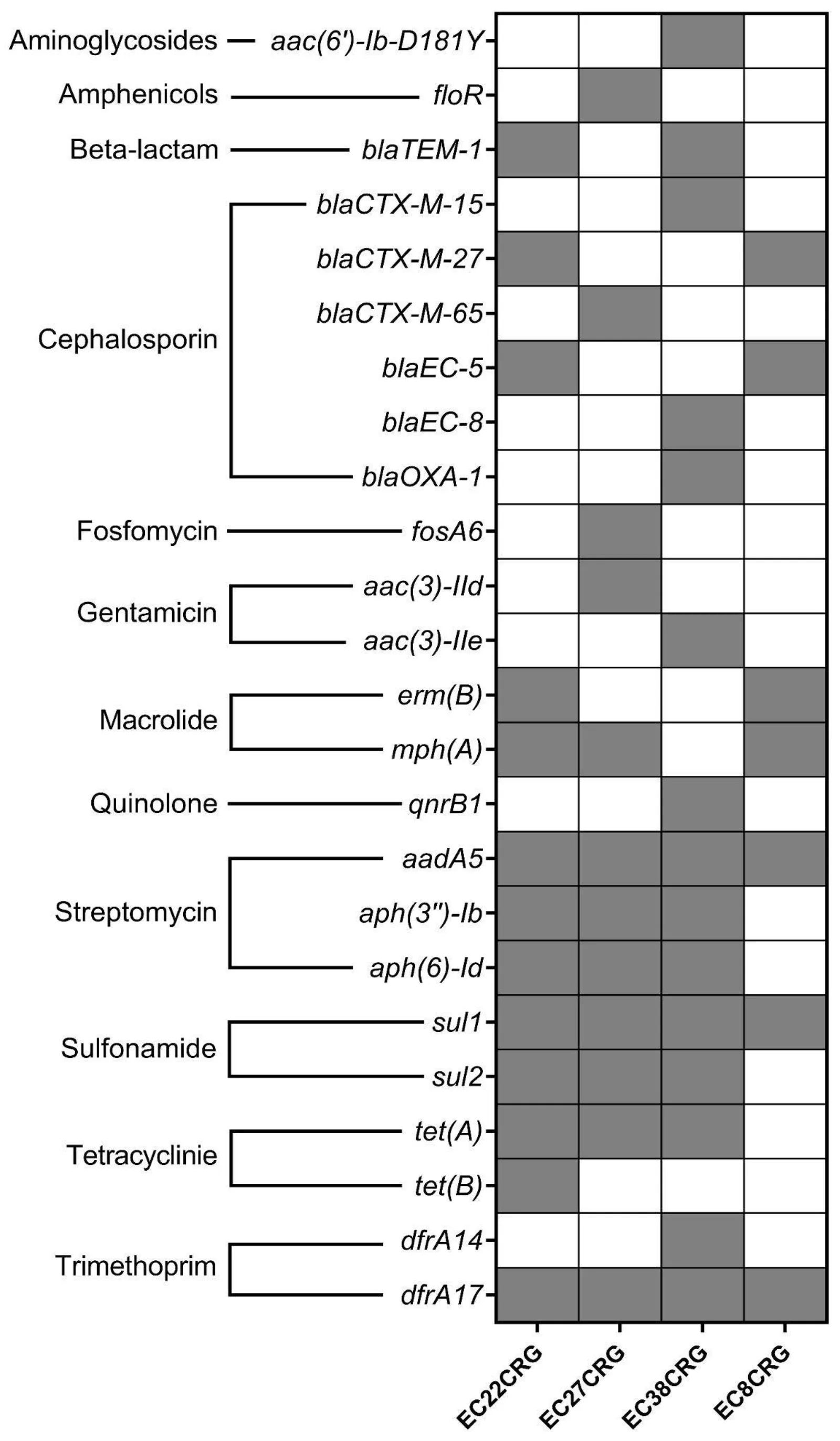
Heatmap of distribution of ARGs across *E*. *coli* clinical isolates. Gray cells indicate the presence of ARGs, whereas white cells indicate their absence.

All four isolates carried extended-spectrum β-lactamase (ESBL) genes, although the specific ESBL-encoding variants differed (Figure 2). EC22CRG and EC8CRG harbored *bla_CTX-M-27_*, EC27CRG carried *bla_CTX-M-65_*, and EC38CRG contained *bla_CTX-M-15_* together with *bla_OXA-1_*, *bla_TEM-1_* was identified in EC22CRG and EC38CRG. Additional acquired ARGs included aminoglycosides (*aadA5*, *aph(3″)-Ib*, *aph(6)-Id*, *aac(3)-IId*, *aac(6′)-Ib-D181Y*), fluoroquinolones (*qnrB1*), tetracyclines (*tet(A)*, *tet(B)*), sulfonamides (*sul1*, *sul2*), trimethoprim (*dfrA14*, *dfrA17*), macrolides (*mph(A)*, *erm(B)*), phenicols (*floR*), and fosfomycin (*fosA6*).

Chromosomal mutations associated with quinolone resistance were detected in three isolates (Supplementary Table S3). Substitutions in GyrA (S83L, D87N) and ParC (S80I) were found in EC27CRG. EC22CRG harbored ParC (S80I) along with ParE (L416F), whereas EC8CRG presented ParE (L416F) as the only resistance-associated substitution detected by PointFinder. No chromosomal resistance-associated mutations were identified in EC38CRG.

#### 2.2.2. Virulome

A total of 92 VFs were identified across the four isolates. EC8CRG exhibited the largest number of virulence-associated genes (*n* = 72), whereas EC27CRG showed the fewest (*n* = 55), with EC22CRG and EC38CRG displaying intermediate profiles. These genes were classified into the following categories: metabolic systems, iron acquisition mechanisms, adhesins, invasins, effector delivery systems, and enterotoxins (Supplementary Table S4).

**Figure 3:**
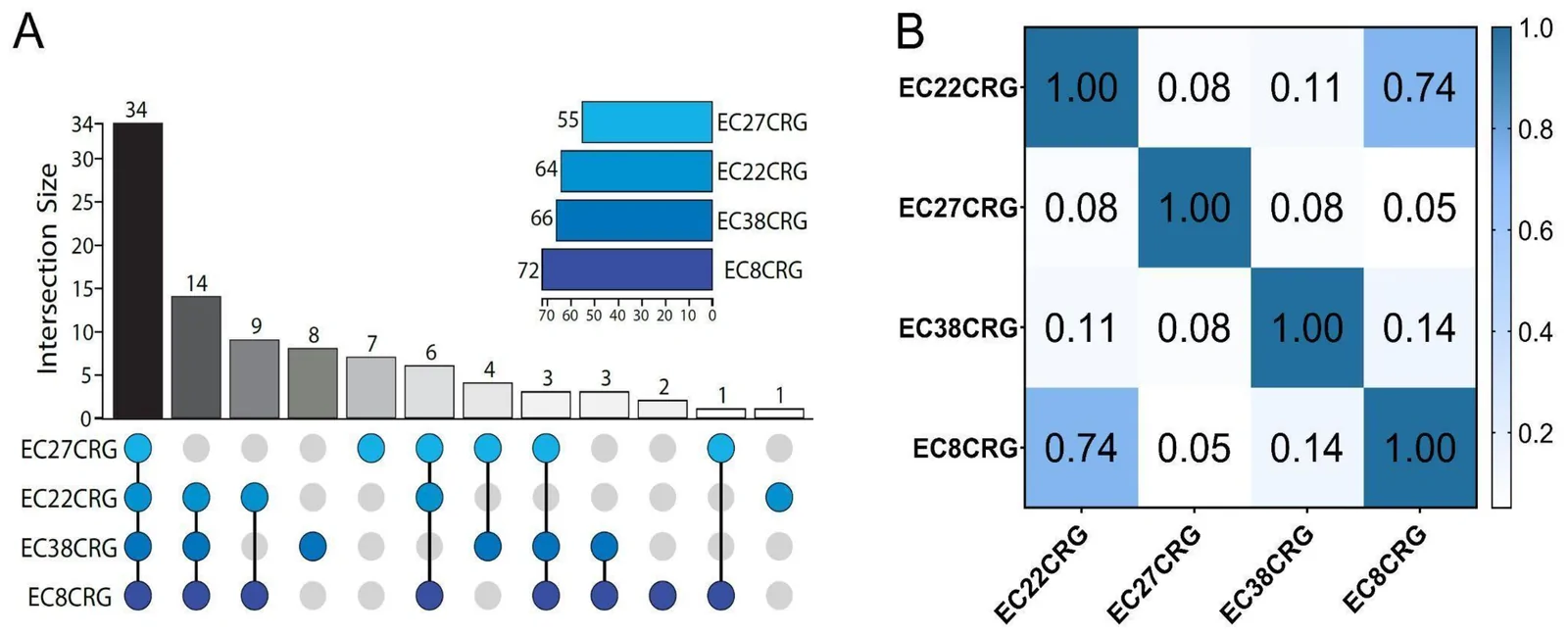
Comparative analysis of VF profiles among the four *E. coli* isolates. (**A**) UpSet plot showing shared and unique VFs. Horizontal bars indicate the total number of VFs detected in each isolate. (**B**) Pairwise Pearson correlation matrix based on VF presence-absence profiles.

34 virulence-associated genes were shared by all four isolates, whereas additional genes showed isolate-specific or partially shared distributions (Figure 3A). EC8CRG had the highest number of unique VFs. EC8CRG contained the largest number of isolate-specific virulence-associated genes. Pairwise comparison of the presence–absence profiles showed the greatest similarity between EC22CRG and EC8CRG (r = 0.74), whereas EC27CRG showed weak correlations with the remaining isolates (r = 0.05–0.08) (Figure 3B).

**Figure 4:**
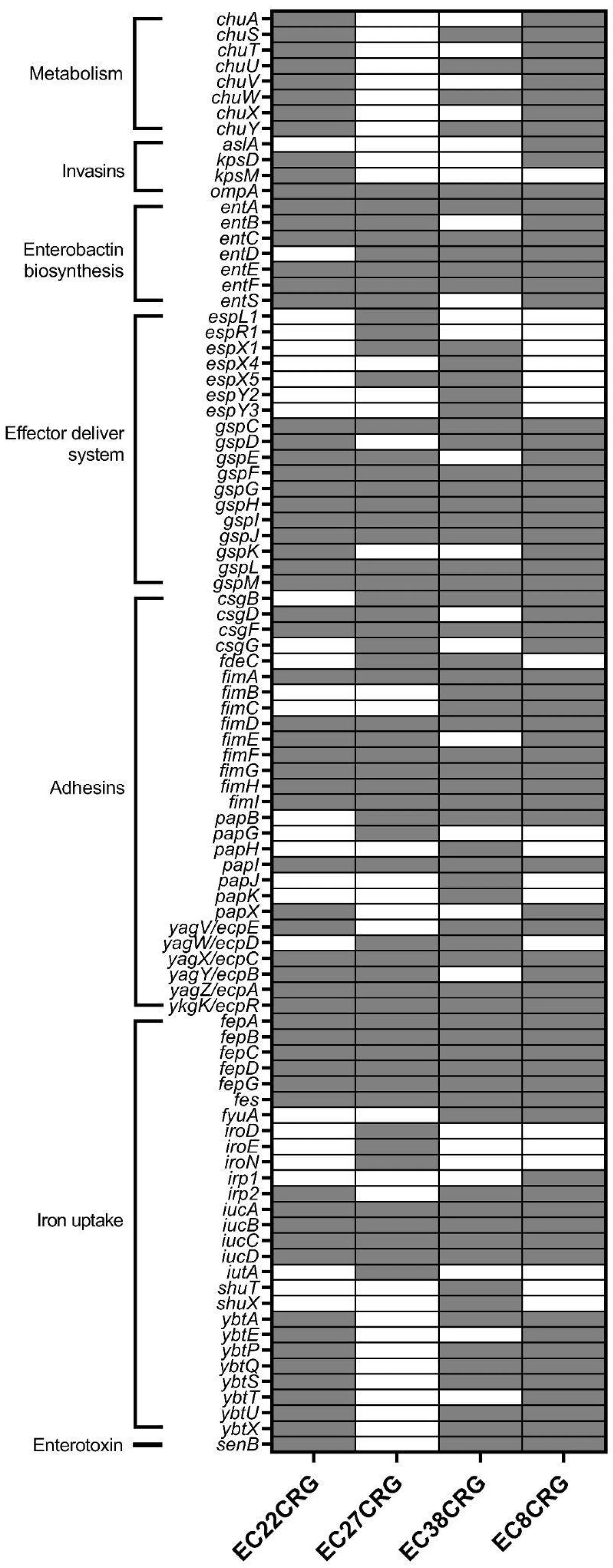
Heatmap showing presence–absence of VFs across the four isolates. Gray cells indicate the presence of VFs, whereas white cells indicate their absence. VFs are grouped according to their predicted functional categories.

Genes assigned to metabolic and iron acquisition-related functions were broadly represented across the isolates, although their distribution varied among strains (Figure 4). The ferrienterobactin transport genes *fepA*, *fepB*, *fepC*, *fepD*, and *fepG*, together with *fes*, were detected in all four isolates, whereas genes of the enterobactin biosynthesis system showed a broad but non-uniform distribution. The heme uptake *chu* system also differed among isolates: EC22CRG and EC8CRG harbored the complete set of *chuA*, *chuS*, *chuT*, *chuU*, *chuV*, *chuW*, *chuX*, and *chuY* detected in this analysis, whereas EC38CRG contained a partial *chu* repertoire and EC27CRG lacked the detected *chu* genes. Additional iron acquisition determinants associated with salmochelin (*iroD*, *iroE*, *iroN*), yersiniabactin (*irp1*, *irp2*, *ybtA*, *ybtE*, *ybtP*, *ybtQ*, *ybtS*, *ybtT*, *ybtU*, *ybtX*, and *fyuA*), and aerobactin (*iucA–iucD*, *iutA*) showed differential distributions among isolates.

Adhesion-associated genes were also widely represented. Type 1 fimbrial genes (*fimA–fimI*) were broadly distributed, together with P fimbriae-associated genes (*papB*, *papG*, *papH*, *papI*, *papJ*, *papK*, and *papX*) and *E. coli* common pilus genes (*yagV/ecpE*, *yagW/ecpD*, *yagX/ecpC*, *yagY/ecpB*, *yagZ/ecpA*, and *ykgK/ecpR*). The adhesin *fdeC* was also detected. Additional virulence-associated genes included *aslA*, the capsule-associated genes *kpsD* and *kpsM*, *ompA*, Type III secretion system-associated effectors (*espR1*, *espX1*, *espX4*, *espX5*, and *espY2*), and general secretion pathway components (*gspC–gspM*), with variable distributions across the four isolates. The enterotoxin-associated gene *senB* was detected in EC22CRG, EC38CRG, and EC8CRG but was absent from EC27CRG.

**Figure 5:**
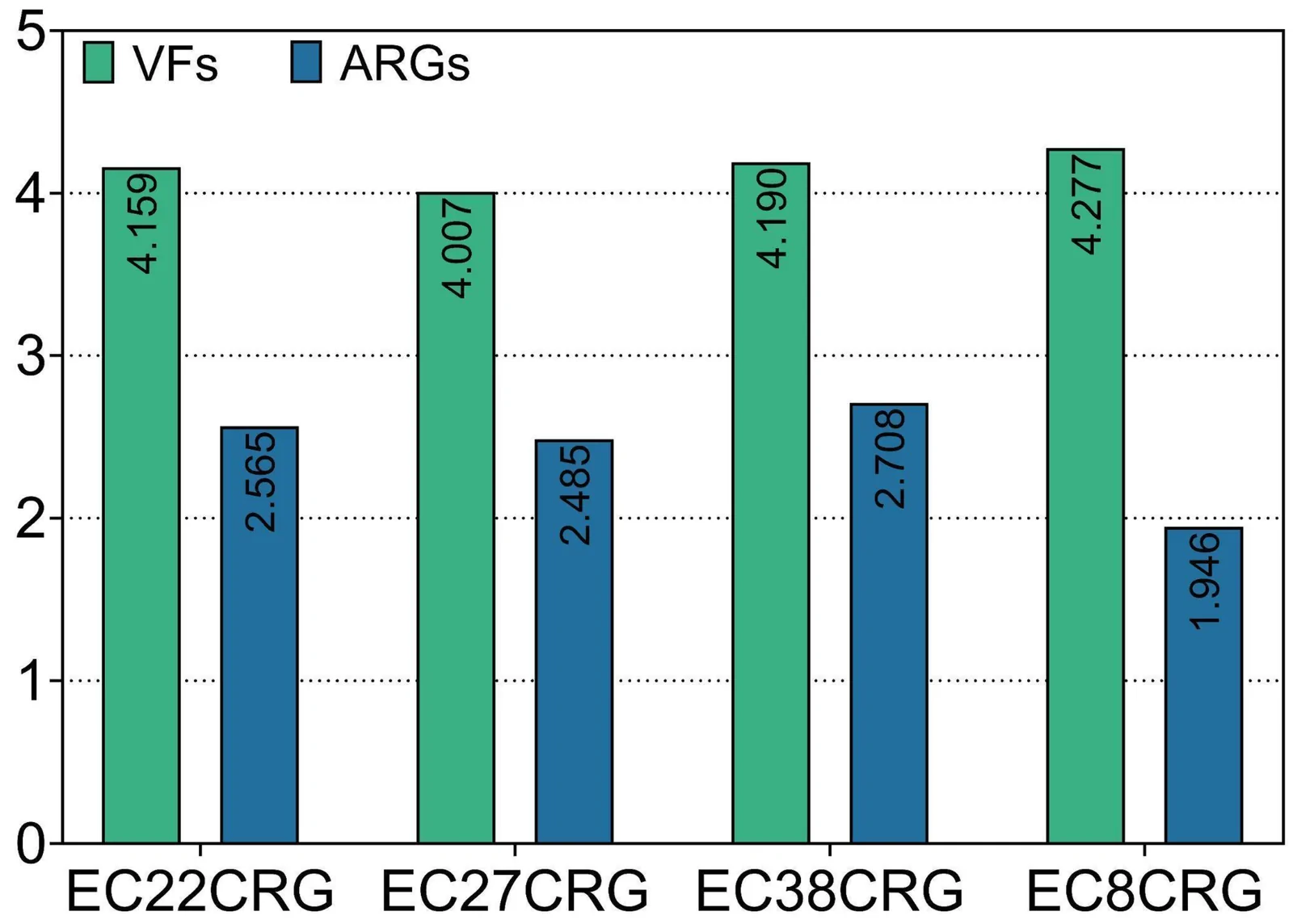
Comparative Shannon diversity indices of VFs and ARGs among clinical *E. coli* isolates.

The Shannon index was used to evaluate the diversity of VFs and ARGs (Figure 5). VF profiles showed greater Shannon diversity than ARG profiles across the isolates, reflecting a broader range of VFs. This greater diversity should not, however, be taken as evidence of increased virulence. The presence of these genes alone cannot predict their phenotypic contribution, which depends on their expression and regulation and on the conditions encountered during host infection. Although the differences in diversity were not statistically significant for either VFs or ARGs, EC8CRG showed the highest VF diversity and the lowest ARG diversity. EC38CRG registered lower VF diversity than EC8CRG but had the highest ARG diversity among the isolates.

#### 2.2.3. MGEs

Analysis using MobileElementFinder predicted a diverse repertoire of MGEs across the sequenced isolates, but only a subset of identified elements was co-localized with ARGs (Supplementary Table S5). IS6100 was detected in all four isolates. EC8CRG, EC22CRG, EC27CRG, and IS6100 occurred on contigs containing the resistance gene cluster *dfrA17–aadA5–sul1–mph(A)*, associated with resistance to trimethoprim, streptomycin, sulfonamides, and macrolides, respectively. In contrast, IS6100 in EC38CRG occurred on a contig containing *dfrA14*.

Additional isolate-specific MGE–ARG associations were observed. In EC27CRG, multiple MGEs, including *ISVsa5*, *IS102*, and *ISEc30*, were detected on contigs that also contained ARGs such as *aac(3)-IId* (gentamicin resistance), *bla_CTX-M-65_* (cephalosporin resistance) and *sul2* (sulfonamide resistance). Similarly, EC38CRG had three MGEs (*ISEc9, IS5075*, and *IS102*) that were co-localized on the same contig as *bla_CTX-M-15_* and *bla_TEM-1_* (beta-lactam resistance), *aph(6)-Id* and *aph(6)-Ib* (streptomycin resistance), and *sul2* (sulfonamide resistance). Additionally, the transposon *Tn5403* was also identified in EC38CRG on contig k141_167, which additionally contained *tet(A)* (tetracycline resistance) and *qnrB1* (quinolone resistance) genes.

Furthermore, plasmid replicon analysis identified several profiles among the isolates. EC8CRG carried Col(BS512), Col156, IncFIA, and rep6; EC22CRG carried Col(BS512), IncFIA, and IncFII(pHN7A8); EC27CRG carried IncFIB(AP001918) and EC38CRG harbored Col156, IncFIB(K), IncFII(29), and IncQ1. IncF-family replicons were therefore detected in all four isolates, whereas Col-type replicons were identified in all except EC27CRG (Supplementary Table S5).

#### 2.2.4. Phylogenetic Context

**Figure 6:**
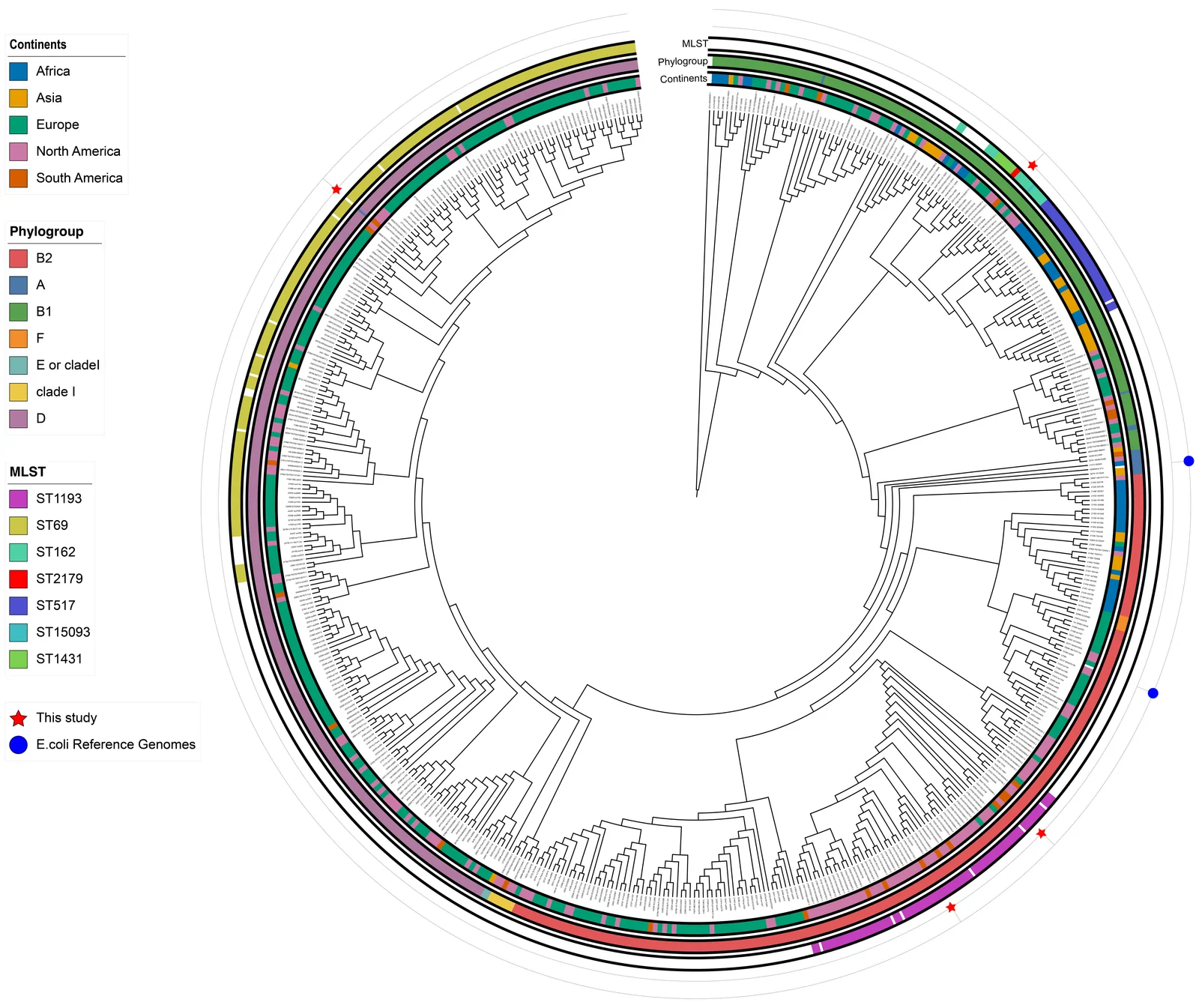
MASH distance-based phylogenetic tree of 548 *Escherichia coli* genomes, including the four pediatric urinary isolates sequenced in this study. Circular phylogenetic tree constructed with Mashtree based on MASH distances and rooted with *Bacillus subtilis* (NCBI accession: GCA_000009045.1) as an outgroup. Concentric metadata rings represent, from innermost to outermost: (1) geographic origin; (2) Phylogroups; and (3) Sequence Types (MLST). Red stars indicate the four genomes sequenced in this study, whereas blue circles indicate reference *E. coli* genomes included for comparative context.

Phylogenomic reconstruction revealed that the four isolates in this study clustered closely with global reference strains, particularly those from South America, North America, and Europe (Figure 6). The MASH distance-based phylogenetic tree showed that EC8CRG and EC22CRG clustered with representative ST1193 genomes within phylogroup B2, corroborating their initial MLST prediction. Isolate EC27CRG, which was assigned to ST162, clustered tightly within a monophyletic branch comprising ST162 strains of phylogroup B1. Notably, EC38CRG isolate, whose sequence type could not be resolved by standard MLST scheme, exhibited high genomic proximity to ST69 lineages. Although EC38CRG was assigned to phylogroup A, it was nested within a cluster dominated by phylogroup D genomes. Overall, the expanded whole-genome comparison supported the ST1193 assignments of EC8CRG and EC22CRG and the ST162 assignment of EC27CRG, while providing additional genomic context for the unresolved EC38CRG isolate. Phylogenetic placement was interpreted as lineage-level genomic context and was not used independently to infer uropathogenicity.

## 3. Discussion

This exploratory study provides a whole-genome characterization of pediatric urinary *Escherichia coli* isolates recovered at a tertiary pediatric hospital in Guayaquil, Ecuador. WGS revealed substantial heterogeneity in sequence types, phylogroups, resistance determinants, mobile genetic elements, plasmid replicons, and virulence-associated gene profiles. By integrating genomic analysis with available antimicrobial susceptibility testing, this study provides an initial genomic description of pediatric urinary *E. coli* isolates recovered in this clinical setting and highlights the value of WGS for understanding the dissemination of clinically relevant lineages, antimicrobial resistance determinants, and virulence traits.

EC22CRG and EC8CRG isolates were assigned to *ST1193*, a globally disseminated, high-risk extraintestinal clone within phylogroup B2. This lineage has spread rapidly worldwide and is strongly associated with fluoroquinolone resistance and the presence of ESBL genes, particularly *bla_CTX-M-27_* and *bla_CTX-M-55_* [11]. Consistent with these reports, both ST1193 isolates characterized in this study exhibited resistance profiles typical of clinically relevant B2 lineages, supporting the presence of clinically relevant resistance features in these isolates. In contrast, EC27CRG belonged to *ST162* within phylogroup B1, which has a broader ecological distribution. This ST has been reported in humans, animals, and environmental reservoirs and is often described as a “bridge lineage” that facilitates the exchange of ARGs across compartments [21]. Although ST162 is less frequently associated with ExPEC infections than the B2 lineage, its identification in a pediatric clinical setting highlights the potential value of continued genomic surveillance of AMR, mainly on a One Health approach given the limited genomic characterization of ExPEC isolates in pediatric urinary isolates from Ecuador [22]. The isolates also represented phylogroups B2, B1, and A, all of which have been reported among UTI-associated *E. coli*, although B2 and D are more frequently represented in UPEC collections [23,24]. Although EC38CRG was assigned to phylogroup A by ClermontTyping, it was positioned within a phylogenetic cluster dominated by phylogroup D genomes, a type of discrepancy that has been described between marker-based phylogroup assignment and whole-genome analyses and may be influenced by recombination [25,26].

Despite the phylogenetic heterogeneity among isolates, all strains shared a conserved virulence backbone, particularly determinants involved in adhesins and iron acquisition systems. Type 1 and P fimbriae-associated genes, capsule-associated determinants, and multiple iron acquisition systems were variably represented among the four genomes. Several of these features have been widely described in UPEC and other ExPEC lineages and are implicated in colonization and persistence during extraintestinal infection [27]. However, UPEC is genetically heterogeneous, and no single virulence-associated gene repertoire unequivocally defines the pathotype. In contrast, the resistome exhibited greater variability among isolates. This observation likely reflects the strong selective pressures imposed by antimicrobial exposure, which drive the acquisition, diversification, and turnover of resistance determinants in horizontal gene transfer, plasmid exchange, and recombination [28]. The predicted association of ARGs with MGEs, including *IS6100* and *IncF* plasmid replicons, suggests the presence of genomic features potentially involved in resistance gene mobility.

Among the isolates analyzed, EC8CRG harbored the most extensive virulence repertoire (72 VFs) but the fewest ARGs. Given the limited number of isolates analyzed, this observation cannot establish a virulence–resistance trade-off. However, the observed genomic pattern is compatible with this previously proposed hypothesis [29,30]. Highly virulent lineages with relatively limited acquired resistance determinants may still cause severe infections, emphasizing that antimicrobial susceptibility does not necessarily predict reduced pathogenic potential. However, recent evolutionary studies have shown that compensatory mutations can rapidly mitigate the fitness cost of plasmid carriage, allowing VFs and ARGs to coexist and remain stably present in clinical strains [31]. This combination represents a particularly concerning scenario in pediatric populations, where therapeutic options are already limited. Our exploratory analysis pointed to a possible difference in VF burden between phylogroup B2 (EC8CRG) and B1 (EC27CRG) isolates. However, given the small number of isolates examined, this pattern should be interpreted with caution. A larger and more representative set of isolates will be needed to determine whether the observed differences are consistently associated with phylogenetic background.

The predicted occurrence of *bla_CTX-M-15_* and *bla_CTX-M-65_* in genomic contexts containing insertion sequences such as IS102, ISEc30, and ISEc9 is consistent with the known involvement of insertion sequences in the dissemination of ESBL determinants [17,28]. However, the fragmented assemblies can lead to conflicting reconstructions of complete genomic regions and therefore do not allow specific mobilization elements to be inferred. The presence of *bla_CTX-M-15_* in our isolates is consistent with previous reports from Ecuador that describe this determinant in both clinical and environmental *E. coli*, which is frequently associated with high-risk ExPEC lineages [32,33]. Likewise, the coexistence of *bla_CTX-M-15_, bla_TEM-1B_,* and *bla_OXA-1_* observed in this study reflects β-lactam resistance profiles commonly reported among internationally disseminated multidrug-resistant ExPEC lineages [34], suggesting that similar multidrug-resistant genomic configurations are present in the local setting.

Beyond beta-lactam resistance, the isolates carried clinically relevant determinants associated with aminoglycosides and fluoroquinolones, indicating an MDR genetic background of these pediatric urinary *E.coli* isolates. The co-occurrence of *aac(6′)-Ib-D181Y* and *qnrB1* is consistent with resistance profiles frequently described in MDR ExPEC lineages, where reduced susceptibility to fluoroquinolones and aminoglycosides often accumulates through the combined acquisition of plasmid-mediated determinants and chromosomal mutations [35,36]. In isolate EC38CRG, the simultaneous presence of these determinants suggests the potential for multidrug-resistant phenotypes driven by multiple complementary resistance mechanisms. Notably, the co-occurrence of *bla_CTX-M-15_*, *bla_OXA-1_*, *aac(6′)-Ib-D181Y*, and qnrB1 in a subset of isolates indicates a genetic configuration frequently associated with plasmid-mediated multidrug resistance involving multiple antibiotic classes [37]. Plasmid replicon analysis revealed multiple incompatibility groups, predominantly belonging to the IncF family. These conjugative plasmids are globally recognized as major vehicles for the horizontal transfer of ARGs [38,39,40]. Concurrently, except for EC27CRG, Col-type replicons were detected in all isolates. Col plasmids encode colicins, narrow-spectrum antimicrobial proteins capable of inhibiting closely related competing bacteria [41]. By reducing competition over resident commensal bacteria, these bactericins may facilitate early colonization and persistence within the gastrointestinal reservoir before urinary tract infection [42]. Ultimately, the co-occurrence of conjugative IncF plasmids and Col-type replicons in these clinical isolates may provide a dual biological advantage by facilitating both the dissemination of ARGs and successful bacterial persistence within intestinal and urinary tract niches.

Complementing the plasmid profiles, analysis of MGEs identified specific insertion sequences and transposons located in genomic contexts containing ARGs, suggesting predicted genomic associations. Notably, the insertion sequence *IS6100* was detected in all four isolates. This element was consistently identified in genomic contexts containing a conserved multidrug resistance cluster comprising *dfrA17*, *aadA5*, *sulI*, and *mph(A)*, suggesting a predicted genomic association with this resistance cluster, which has been associated with the assembly and dissemination of complex resistance integrons [39]. Beyond the conserved *IS6100*-associated resistance cluster, additional insertion sequences were identified in association with ESBL genes, illustrating the diversity of genetic contexts surrounding these resistance determinants. The predicted genomic association of *bla_CTX-M-65_* and *bla_CTX-M-15_* with multiple insertion sequences, such as *IS102*, *ISEc30*, and *ISEc9*, suggests that these resistance determinants may occur within mobile genetic contexts. *ISEc9* and *IS102* have been reported to promote genetic plasticity and to participate in the mobilization of *bla_CTX-M_* in clinical Enterobacteriaceae [35]. In particular, *ISEc9* has been reported to harbor an outward-facing promoter capable of enhancing *bla_CTX-M_* expression, a mechanism associated with increased β-lactam resistance [36]. Additionally, *qnrB1* and *tet(A)* were identified in a genomic context predicted to be associated with the transposon *Tn5403*. This transposon has been previously reported to contribute to the mobilization and co-occurrence of resistance determinants [43]. These findings indicate that the pediatric urinary *E. coli* isolates analyzed harbor diverse genomic contexts associated with multidrug resistance. Mobile genetic elements may contribute to the acquisition and dissemination of resistance determinants [44]. However, the present analysis does not establish physical linkage or mobilization events.

Analysis of the chromosomal quinolone resistance-determining regions (QRDRs) revealed distinct mutation profiles among the isolates. EC27CRG exhibited the most extensive QRDR profile, harboring canonical GyrA S83L/D87N and ParC S80I substitutions. EC22CRG and EC8CRG carried mutations in ParE (L416F) and ParC (S80I). These substitutions have been consistently associated with reduced fluoroquinolone susceptibility in UPEC through decreased quinolone binding affinity to DNA gyrase and topoisomerase IV [45]. The presence of the ParC S80I substitution was identified in multiple isolates and was assigned a high deleteriousness score by MutPred2 (0.883), suggesting a potential impact on protein function [46]. Previous functional studies have shown that this substitution reduces fluoroquinolone binding to topoisomerase IV, thereby contributing to decreased susceptibility [47]. In contrast, EC38CRG lacked chromosomal QRDR mutations, although it carried the plasmid-mediated quinolone resistance determinant qnrB1. Overall, the genomic resistance profiles showed substantial agreement with the available phenotypic susceptibility data across several antimicrobial classes, supporting the potential utility of WGS as a complementary tool for antimicrobial resistance prediction (Supplementary Table S1) [48]. However, incomplete antibiogram data from the hospital laboratory limited validation for certain antibiotic classes.

This study has several limitations. Most notably, the analysis was based on only four isolates collected from a single pediatric hospital over a brief period in 2020. Therefore, the observed sequence types, resistance determinants, virulence-associated genes, and mobile genetic elements should not be considered representative of pediatric urinary *E. coli* epidemiology in Ecuador. Additionally, due to the fragmented nature of the draft genome assemblies, the precise co-localization of resistance genes on specific mobile genetic elements (such as plasmids or transposons) could not be conclusively determined; thus, these associations represent co-occurrence rather than physical linkage. Finally, virulence-associated genes were identified by genomic prediction and were not evaluated experimentally for expression or functional contribution. Larger, multicenter studies integrating detailed clinical metadata, standardized antimicrobial susceptibility testing, phenotypic virulence assays, and long-read sequencing will be needed to validate and extend these findings. Overall, these results offer an exploratory genomic characterization of these particular clinical isolates and underscore the need for larger, multicenter, and longitudinal genomic surveillance studies using long-read sequencing technologies.

## 4. Materials and Methods

### 4.1. Clinical Isolates

From the Enterobacteriaceae collection of the pediatric Roberto Gilbert Hospital biobank, four *E. coli* isolates recovered from urine cultures were randomly selected. The isolates were collected between November and December 2020, during the COVID-19 pandemic. Available clinical records indicated that EC8CRG, EC27CRG, and EC38CRG were recovered from hospitalized pediatric patients admitted to either the intensive care unit (ICU) or general hospital wards; the specific clinical setting from which EC22CRG originated could not be retrieved from the available records. For EC38CRG, sufficient clinical documentation was available to support a diagnosis of urinary tract infection (UTI). For EC8CRG and EC27CRG, the available records documented a positive urine culture and clinical suspicion of UTI but lacked sufficient clinical information to independently confirm the diagnosis. For EC22CRG, only its urinary origin could be confirmed from the available records. Accordingly, the four strains are collectively referred to as pediatric urinary *E. coli* isolates, and uropathogenicity was not inferred solely from urinary origin or the in silico detection of virulence-associated genes. Antimicrobial susceptibility testing was performed for ampicillin (AMP), ampicillin-sulbactam (SAM), cefuroxime (CEF), ciprofloxacin (CIP), gentamicin (GEN), and trimethoprim-sulfamethoxazole (SXT) using the VITEK-2 Gram-Negative Susceptibility Test Cards (AST-GN04, bioMérieux, Marcy-l’Étoile, France). Resistance and susceptibility to each antibiotic were retrospectively interpreted according to the CLSI 2025 guidelines [49].

### 4.2. DNA Extraction and WGS

Genomic DNA was prepared using a QIAamp DNA Kit (Qiagen, Redwood, CA, USA) according to the manufacturer’s instructions. DNA concentration was assessed using a Qubit 4.0 fluorometer (Invitrogen™, Thermo Fisher Scientific, Waltham, MA, USA). The sequencing library was prepared from 100 ng of genomic DNA using a Tagmentation-based library Prep Kit (Illumina, Inc., Foster City, CA, USA) according to the manufacturer’s protocol. Finally, 150 bp paired-end reads were sequenced from the library on an Illumina MiniSeq using the High Output Reagent Kit (Illumina, Inc., Foster City, CA, USA).

### 4.3. Read Quality, Genome Assembly, and Annotation

The quality of the reads was analyzed using the FastQC tool [50]. Reads with a Q-score below 30 were trimmed using Trimmomatic v0.39 [51]. De novo assembly was performed on the clean reads using MEGAHIT v1.2.9 [52], with default parameters. The assemblies were annotated using Bakta v1.10.4 with the database v6.0 [53]. CheckM2 v1.1.0 [54] was used to determine genome completeness and contamination.

### 4.4. ARGs and VFs Detection

The resistome was characterized using a dual approach to ensure comprehensive detection. First, ABRicate v1.0.1 [55] was run against the NCBI AMR FinderPlus database [56]. Default parameters were applied, including a minimum identity threshold of 80% and a minimum coverage of 80% for gene detection. Second, STARAMR v0.11.0 [57] was used to integrate the detection of acquired ARGs (ResFinder) [58], chromosomal point mutations (PointFinder) [59], and plasmid replicons (PlasmidFinder) [60]. Relevant databases were downloaded and updated at runtime using the standard db build function. VFs were identified using ABRicate v1.0.1 with the VFDB database [61]. Detected genes were grouped into key functional categories relevant to urinary tract infections, including adhesins, toxins, siderophores, capsule synthesis genes, and invasins.

### 4.5. Mobile Genetic Elements and Plasmid Replicon Detection

MGEs, such as Insertion Sequences (ISs) and Transposon Elements (TEs), were identified using MobileElementFinder v1.1.2 [62] run within a Docker container (docker run mkhj/mobile_element_finder). Plasmid replicon detection was further validated by comparison with the PlasmidFinder module integrated in STARAMR.

### 4.6. Molecular Typing and Phylogenetic Context

Average nucleotide identity (ANI) was calculated with FastANI v1.34 [63] against the reference genomes K-12 and ST131, selected to provide a well-characterized baseline for comparative genomics and to represent a globally disseminated high-risk clone, respectively. To characterize the phylogenetic identity of the isolates, multilocus sequence typing (MLST) was performed using the MLST tool v2.23.0 [64], which incorporates components of the PubMLST database [65], applying the *E. coli* Achtman MLST scheme. To ensure robustness of sequence type assignments, results were cross-validated using the PubMLST platform. Phylogroups were assigned with ClermonTyping v24.02 [66], and O:H serotypes were predicted using ECTyper v0.8.1 [67].

Phylogenetic relationships among isolates and reference genomes were assessed using a whole-genome MASH distance-based approach using Mashtree (v1.4.6) [68]. The dataset comprised a total of 548 genomes, including 527 *Escherichia coli* genomes retrieved from PubMLST, representing Africa (*n* = 47), Asia (*n* = 32), North America (*n* = 156), South America (*n* = 6), and Europe (*n* = 286); 15 Ecuadorian genomes sourced from the NCBI database; 2 additional reference genomes *(E. coli* K-12 MG1655 and UTI89); and the 4 isolates sequenced in this study. All 544 selected external genomes successfully passed quality control and taxonomic confirmation filters evaluated using CheckM2 (v1.1.0) [54] and FastANI (v1.34) [63] against the *E. coli* K-12 MG1655 reference genome (GenBank accession: NC_000913.3), meeting strict thresholds of completeness ≥ 95%, contamination ≤ 5%, and Average Nucleotide Identity (ANI) ≥ 95% (Supplementary Table S6). Well-characterized reference strains, including *E. coli* K-12 MG1655 (GenBank accession: NC_000913.3) and UTI89 (GenBank accession: NC_CP000243.1), were incorporated for comparative baseline analysis. K-12 is a well-characterized laboratory strain whose complete genome annotation serves as a baseline for comparative genomics and functional studies of *E. coli* [69]. Genomes were selected to represent lineages closely related by sequence type (ST) and phylogroup, with a particular focus on globally disseminated high-risk clones such as ST1193, an extraintestinal pathogen associated with multidrug resistance (MDR) and co-resistance to fluoroquinolones and beta-lactams [70,71]. *Bacillus subtilis* (GenBank accession: GCA_000009045.1) was used as an outgroup to root the tree. Tree visualization and annotation were carried out in Interactive Tree of Life (iTOL) v7 [72]. UTI89 is a prototypical UPEC strain isolated from a human urinary tract infection and extensively characterized for virulence mechanisms and bladder colonization dynamics [73]. Their inclusion enables phylogenetic comparisons across commensal, laboratory, and pathogenic lineages.

## 5. Conclusions

The study provides a WGS characterization of pediatric urinary *E. coli* isolates recovered at a tertiary pediatric hospital in Guayaquil, Ecuador. The isolates reveal a conserved VF repertoire among distinct STs together with diverse antimicrobial resistance determinants, plasmid replicons, mobile genetic elements, and chromosomal QRDR mutations. The identification of the globally disseminated high-risk clone ST1193 lineage carried *bla_CTX-M-27_*, whereas another isolate belonged to ST162. These findings provide initial genomic evidence of clinically relevant multidrug-resistant *E. coli* lineages among pediatric urinary isolates from this hospital and highlight the value of integrating WGS, phenotypic antimicrobial susceptibility testing, and clinical metadata in larger-scale surveillance studies.

## Figures and Tables

**Table 1:** Summary of molecular typing results and ANI values (%) of study isolates against reference genomes.

Features	EC22CRG	EC27CRG	EC38CRG	EC8CRG
Assembly Length (Mbps)	5.0	4.9	5.1	4.8
N50 (Kbps)	6.7	8.4	4.6	8
Contigs	1328	1146	1929	1318
ST	1193	162	ND	1193
Phylogroup	B2	B1	A	B2
Serotype	O75:H5	H51	O153:H2	O75:H5
ANI with ST131	99.2	98.6	98.7	99.1
ANI with K-12	98.6	98.5	99.1	98.5
Completeness	95.04	98.34	95.11	98.56
Contamination	1.31	0.49	1.76	3.05

## Data Availability

The *E. coli* WGS data will be deposited in the public archive of NCBI GenBank BioProject under accession number PRJNA1293794.

## Supplementary Materials

The following supporting information can be downloaded at: https://www.mdpi.com/article/10.3390/antibiotics15100996/s1, Table S1. Clinical and phenotypic characteristics of pediatric urinary *E. coli* isolates from a tertiary hospital in Guayaquil, Ecuador. Table S2. Multilocus sequence typing (MLST) allelic profiles and sequence type assignments for the clinical *E. coli* isolates. Table S3. Chromosomal mutations and predicted resistance phenotypes in *E. coli* isolates. Table S4. VF categories and distribution across *E. coli* isolates analyzed in this study. Table S5. Distribution and genomic localization of MGEs identified in *E. coli* clinical isolates. Table S6. PubMLST and NCBI Isolates of *E.coli* used for MASH Distance Tree inference.

## Abbreviations

UPEC	Uropathogenic *Escherichia coli*
VFs	Virulence factors
ARGs	Antimicrobial resistance genes
UTIs	Urinary tract infections
ExPEC	Extraintestinal pathogenic *E. coli*
STs	Sequence types
WGS	Whole genome sequencing
SAM	Ampicillin-sulbactam
CEF	Cefuroxime
CIP	Ciprofloxacin
GEN	Gentamicin
AMP	Ampicillin
SXT	Trimethoprim-sulfamethoxazole
MLST	Multilocus sequence typing
